# Deep-reaching thermocline mixing in the equatorial pacific cold tongue

**DOI:** 10.1038/ncomms11576

**Published:** 2016-05-12

**Authors:** Chuanyu Liu, Armin Köhl, Zhiyu Liu, Fan Wang, Detlef Stammer

**Affiliations:** 1 Institute of Oceanography, Center for Earth System Research and Sustainability (CEN), University of Hamburg (UHH), Hamburg 20146, Germany; 2 Key Lab of Ocean Circulation and Waves (KLOCAW), Institute of Oceanology, Chinese Academy of Sciences (IOCAS), Nanhai Road 7, Qingdao 266071, China; 3 Function Laboratory for Ocean and Climate Dynamics, Qingdao National Laboratory for Marine Science and Technology (QNLM), Qingdao 266237, China; 4 State Key Laboratory of Marine Environmental Science (MEL) and Department of Physical Oceanography, College of Ocean and Earth Sciences, Xiamen University, Xiamen 361102, China

## Abstract

Vertical mixing is an important factor in determining the temperature, sharpness and depth of the equatorial Pacific thermocline, which are critical to the development of El Ninõ and Southern Oscillation (ENSO). Yet, properties, dynamical causes and large-scale impacts of vertical mixing in the thermocline are much less understood than that nearer the surface. Here, based on Argo float and the Tropical Ocean and Atmosphere (TAO) mooring measurements, we identify a large number of thermocline mixing events occurring down to the lower half of the thermocline and the lower flank of the Equatorial Undercurrent (EUC), in particular in summer to winter. The deep-reaching mixing events occur more often and much deeper during periods with tropical instability waves (TIWs) than those without and under La Niña than under El Niño conditions. We demonstrate that the mixing events are caused by lower Richardson numbers resulting from shear of both TIWs and the EUC.

## Abstract (web summary)

The role of thermocline mixing in the Pacific cold tongue in the propagation of ENSO remains uncertain. Here, Liu *et al*. show a persistent occurrence of mixing events in the middle and lower parts of the thermocline, particularly during tropical instability waves and under La Niña conditions.

## Graphical abstract

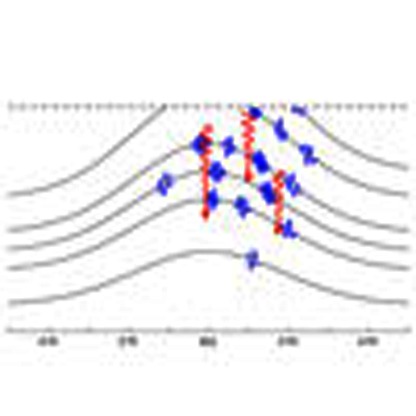

The maintenance of the equatorial Pacific thermocline relies either on high-latitude buoyancy forcing or on extratropical wind and buoyancy forcing^1^,^2^ at annual to inter-annual time scales, but is modulated by local Kelvin waves^3^ and wind stress curl^4^ at intra-seasonal to seasonal time scales. Numerical experiments suggest that the sharpness and depth of the thermocline is also determined by vertical mixing within it^5^,^6^. Measurements and model studies suggests that turbulence and mixing below the mixed layer base of the equatorial Pacific are attributed to the vertical velocity gradient (shear) between the eastward flowing Equatorial Undercurrent (EUC) and the westward flowing South Equatorial Current^7^,^8^,^9^,^10^,^11^,^12^,^13^,^14^,^15^, which is likely to be further modulated by the wind stress^16^,^17^,^18^. Mixing or instabilities in layers further below, ranging from the upper^13^ to the lower^14^,^19^ parts of the thermocline, are also observed from limited measurements. The instabilities in the lower part of the thermocline may be caused by absorption and saturation of wave energy at critical levels^19^, whereas the mixing in the upper part of the thermocline is found to be related to baroclinic inertial-gravity waves^20^, Kelvin waves^14^ and, in particular, the tropical instability waves (TIWs)^13^.

TIWs refer to energetic meanders frequently emerging in the middle and eastern equatorial ocean. They have long been proposed to be a combination of a Yanai(-like) wave on the Equator and a first-meridional-mode Rossby wave just north of the Equator, with periods of 12–40 days and wavelengths of 700–1,600 km (refs 21, 22, 23, 24, 25, 26). Alternatively, TIWs are also suggested to be manifestations of tropical vortices or highly nonlinear waves^27^,^28^.

A prominent feature of TIWs is the large meridional velocity ranging from the surface to the core of the EUC, providing the potential for vigorous interactions with the already energetic equatorial current system. Turbulence measurements taken by a Lagrangian float encountering a TIW^29^ and modelling studies of the impact of TIWs^30^ in the eastern/middle equatorial Pacific both found strong vertical mixing at the base of the surface mixed layer, which induces intensive cooling of the sea surface^30^,^31^. The measurements^29^ suggest that the strong mixing can be explained by the enhancement of shear modulated by the TIW^29^. Direct turbulence measurements at 0°, 140° W encountering a TIW further confirmed the enhancement of mixing by TIW both in and below the surface mixed layer; in particular, the measurements also revealed a tenfold increase in turbulent heat flux in the upper half of the thermocline^13^. The resulting mixing was accompanied with a significant temperature change in the upper 150 m within a cycle of the TIW^32^. The vigorous deep-reaching mixing are also attributed to additional shear provided by the meridional velocity of the TIW above the EUC core^13^.

If this identified relationship between TIW and enhanced deep thermocline mixing is largely representative, it implies that after a long duration of TIWs the associated thermocline mixing may have the potential to alter the structure of the thermocline and the subsurface temperature of the Pacific cold tongue, which may further have an impact on the large-scale oceanic-atmospheric dynamics, such as El Niño and Southern Oscillation (ENSO)^6^ and the global climate at large^33^.

However, observational evidence for the link between the TIWs and enhanced thermocline mixing is far from adequate. To date, direct turbulence measurements were confined to a few specific locations and covered only short time spans. Whether the thermocline mixing is organized in seasonal or longer-period cycles that are mechanistically related to variations of TIWs at the same periods and to what depths the TIWs may have an impact on the vertical mixing need to be explored.

Two databases could be employed to investigate the both issues. One is the Argo float database^34^. More than 3,000 freely drifting Argo floats continuously provide millions of profiles of temperature and salinity in the upper ∼2,000 m ocean. The measurements offer a great opportunity to shed light on vertical mixing, because many profiles possess fine resolution, that is, small enough sample spacing (*O*(1 m)), to resolve turbulent mixing processes in the ocean interior. Mapping the global distribution of vertical mixing based on the Argo observations^35^ with a fine-scale parameterization method^36^ has demonstrated the usefulness of Argo measurements in ocean mixing studies. However, such estimation so far has been restricted to extra-equatorial regions below the thermocline due to limitations of the employed method^36^,^37^. Alternatively, the Thorpe method (see Methods and ref. 38) is suitable in the thermocline and could be applied to fine resolution Argo profiles, to detect mixing events in the equatorial thermocline.

The second database is the Tropical Atmosphere and Ocean (TAO) mooring observations^39^. The TAO array has provided continuous and high-quality oceanographic data including velocity, temperature and salinity in the upper 500 m over the last two decades. The method of linear stability analysis (LSA; see Methods and refs 18, 40, 41, 42) is applied to the hourly profiles of density and velocity at a location in the middle equatorial Pacific. This method enables to detect potential instabilities occurring in the thermocline.

From both databases, we obtained large amount of possible mixing events (featured as density overturns and potential instabilities). We show that the mixing events occurred not only in the upper part of the thermocline but also deep down to the centre and lower part of it. We also show that the mixing events occurred more often and much deeper during periods of TIWs and of La Niña conditions because of stronger shear instabilities.

## Results

### Deep-reaching density overturns in Argo float measurements

**Figure 1:**
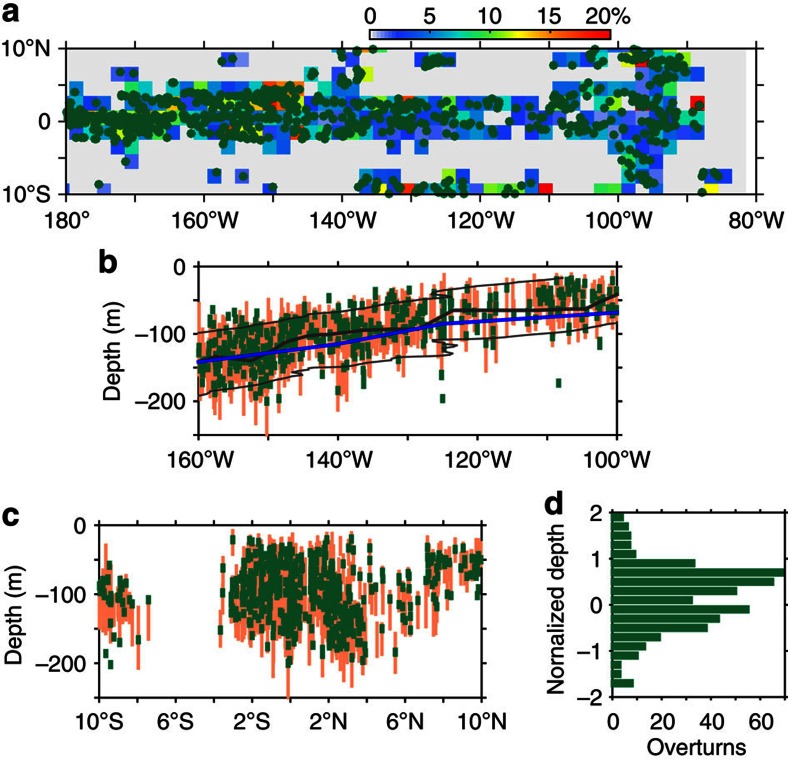
Spatial distribution of detected density overturns in the equatorial Pacific cold tongue. (**a**) Occurrence probability (colour) and horizontal locations of overturns (dark green dots). (**b**) Depth and sizes (in metres) of the overturns (dark green bars) occurred between 3° S and 6° N, and the corresponding PLs (light orange bars) from a latitudinal view. The blue curve denotes the mean depth of the EUC core (averaged over ±1°; data are obtained over the 1990s (ref. 70)). The thick black curve and thin black curves denote the centre (depth of maximum *N*^2^;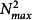) and bounds (depths of half 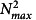) of the mean pycnocline. *N*^2^ is calculated from sample mean density that is meridionally averaged from all fine-resolution Argo profiles over ±1°. (**c**) The same as in **b** but assembled from data between 160 and 100° W (curves of average variables are not added due to large zonal variation). (**d**) Histogram of overturns at the referenced and normalized vertical coordinate.

The equatorial Pacific is a region with accumulated Argo float observations. Among all observed profiles, there exist ∼20,000 fine resolution profiles that are with a sample spacing of no more than 2 m (see Methods) in the upper 200 m and covering 10° S to 10° N and 180 to 80° W over the period of January 2000 to June 2014 (Supplementary Fig. 1). The Thorpe method (see Methods) is applied to the fine resolution profiles and eventually ∼800 density overturns are identified. Among them, a large portion of the overturns occurred after 2008, a period when most of the fine-resolution profiles exist. The horizontal distributions of the detected overturns are shown in Fig. 1a. In particular, in the region between 160° and 100° W, most overturns are confined to the equatorial band, ranging from 3° S to 6° N (about 400 overturns are found between 160°–110° W and 3° S–6° N); east of 100° W, overturns extend meridionally to ±10°. Away from these regions, fewer overturns are detected.

Overlapped by the overturns in Fig. 1a is the occurrence probability of overturns calculated in 2° × 2° bins. The occurrence probability refers to the ratio of the number of Argo profiles that contain overturn(s) to the number of total qualified Argo profiles (see Methods) in a given area. Here, the time span is January 2000–June 2014. The occurrence probability ranges from 1 to 20% and peaks at 3%. It is noteworthy that the small values may not reflect the real occurrence probability of turbulent overturns, because the real overturns may have sizes of 10 cm to several metres, whereas here only the overturns larger than the sample spacing of the Argo profiles (2 m) were detected. Despite the scale selection, the results are still indicative for inferring the relationship between mixing events and TIWs. For example, a prominent feature of the horizontal distribution of the overturns is that they are concentrated in a band across the Equator but display a meridional asymmetry: 3° S–6° N. This band is within the region of the South Equatorial Current and EUC, and matches well the region of the TIWs, which are concentrated at the Equator and have two centres of high temperature variability at 5° N and 2° S (ref. 21).

We further graph the overturns with their corresponding instantaneous pycnocline layers (PLs) against both longitudes (Fig. 1b) and latitudes (Fig. 1c). Here, the centre of a PL is defined as the depth of the maximum *N*^2^ (hereafter 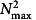, *N*^2^=−g*ρ*_z_/*ρ*_0_ is the buoyancy frequency squared, *ρ*=*ρ*(z) is monotonically sorted potential density and is smoothed by a 40-m running mean, to remove influences of noises or intermittent internal waves, *ρ*_*0*_=1,000 kg m^−3^ is the reference potential density and *g* is the gravitational acceleration). The upper and lower bounds of a PL are defined as the depths where 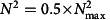 above and below the PL centre (but is additionally bounded by the depth of *N*^*2*^=0.625 × 10^−4^ s^−2^). The size of the most detected overturns is 6 m (Supplementary Fig. 2), whereas the thicknesses of instantaneous PL vary from 50 to 100 m and are generally larger in the western than in the eastern equatorial Pacific (Fig. 1b). The synoptic overturns are confined to the temporally averaged PL (Fig. 1b; thin black curves) of the Equator. It is noticeable that a large fraction occurred below the centre of the average pycnocline (Fig. 1b; the thick black curve); they reached as deep as ∼−200 and ∼−100 m in the western and eastern equatorial Pacific, respectively.

We emphasize that the deep-reaching overturns in the pycnocline in the meantime also reached to the lower flank of the EUC. This can be inferred from Fig. 1b, where the average core of the EUC (the depth of maximum eastward velocity; Fig. 1b; the blue curve) is more or less coincident with the centre of the pycnocline. This result is confirmed in the following by LSA examinations.

Figure 1c shows the meridional distribution of the detected overturns. It confirms the feature that the equatorial overturns are confined between 3° S and 6° N, the regime of the TIWs. Overturns outside this region are mainly found in the region east of 100° W.

The overturns and PLs shown on the physical depths (Fig. 1b,c) are subject to spatial and temporal variations. To provide an overview of the vertical distribution of the overturns relative to their corresponding pycnocline, we redistribute the overturns with respect to a transformed and normalized vertical coordinate (Fig. 1d). This coordinate is referred to the depth of *N*^2^_max_, and normalized in the upper (lower) half of the pycnocline by the thicknesses of the upper (lower) half of each PL. As such, in this coordinate, 0 represents the PL centre, while 1 and −1 represent the upper and lower bounds of the PL, respectively.

It shows that overturns occur not only in the upper part of the PL but also below the centre of the PL. Most overturns occur in the upper three quarters of the PL (between −0.5 and 1). Although in Fig. 1d the overturns peak at ∼0.7, that is, near the upper bound of the pycnocline, it may not mean that the overturns in the ocean really peak here. This is because the prescribed cutoff buoyancy frequency (minimum of *N*^2^=0.5 × *N*^2^_max_ and *N*^2^=0.625 × 10^−4^ s^−2^) in the Thorpe method may have omitted overturns in weak-stratification layers, including the mixed layer and the layer just below. Nevertheless, the overturns peaking at ∼0.7 needs an interpretation. Taking the location 0°, 140° W for reference, the centre and upper bound of the temporally averaged pycnocline are at −100 and −60 m, respectively (Fig. 1b); in consequence, depth 0.7 of the normalized coordinate corresponds to 28 m above the pycnocline centre, that is, at the physical depth of −72 m. According to direct turbulence measurements^13^,^32^, this depth mostly belongs to the upper core layer, which refers to a layer that is located above the EUC core and accompanied with strong TIW-induced turbulence. In the depths above 0.7, the overturns may come from the deep cycle layer^7^,^8^,^10^,^11^,^14^, which refers to a layer several tens of metres below the base of the surface mixed layer that undergoes a nighttime enhancement of turbulence; this layer is dynamically related to the diurnal varying surface buoyancy and wind forcing. It is noteworthy that the TIW-related upper core layer is seemingly separated from the surface-driven deep cycle layer^32^. Between the depths −0.5 and 0.7, more than a half overturns as those at depth 0.7 are found, indicating that intensive turbulence extends into the deep pycnocline.

### Relationship between the deep-reaching overturns and TIWs

**Figure 2:**
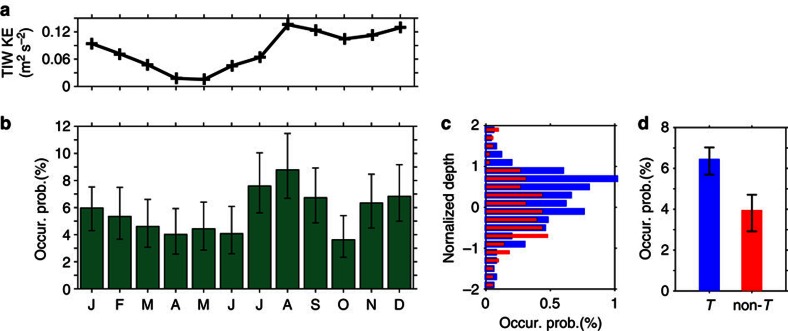
Occurrence probability of detected overturns and its relation to tropical instability waves. (**a**) Monthly climatology of TIWs KE (averaged over years 2005–2011). (**b**) Monthly occurrence probability of overturns between 3° S and 6° N over 160 and 270° W; error bars are 95% bootstrap CIs. Peak at August is significantly different from surrounding troughs in June and October at the 95% bootstrap confidence level; the peak in December is significantly different from the trough at October at the 95% bootstrap confidence level and from troughs in April and June at the 90% bootstrap confidence level. The correlation coefficient between monthly TIWs KE in **a** and occurrence probability in **b** is *r*=0.66, with *P*-value=0.020 and 95% CI=(0.14, 0.89), that is, statistically significant. (**c**) Histogram of the occurrence probability in the normalized coordinate for periods of TIW (blue) and non-TIW (red) (see text). (**d**) Total occurrence probability of overturns during TIW (6.34%, blue) and non-TIW (3.76%, red) periods; error bars are 95% bootstrap CIs (blue: (5.67%, 7.07%) and red: (2.92%, 4.71%)). In **b**,**c** and **d**, the occurrence probability is calculated over the years 2005–2013.

In general, TIWs are active from boreal summer to winter, while inactive in boreal spring^26^. To investigate whether the occurrence of overturns is also organized in such a seasonal cycle, the monthly occurrence probability of overturns in the region of active TIWs, 3° S and 6° N, and 160° W and 100° W, is calculated over the period between January 2005 and December 2013 (Fig. 2b). It is shown that the occurrence probability is indeed subject to similar seasonal variation: they peak in August and December, and have minimum values in boreal spring (April to June) and October. This seasonality is statistically significant. The maximum in August is twice the minima in October and April; the secondary maximum in December is >50% larger than the minima. From direct turbulence measurements over a 6-year span at 0°, 140° W, the vertical heat flux in the subsurface layers (−60 to −20 m) is found to be largest in boreal August, second largest in December, and least in spring and second least in October and November^43^, consistent with the occurrence probability of overturns shown here. Specifically, the monthly occurrence probability is significantly correlated with the multi-year (2005–2010) averaged monthly TIW kinetic energy (KE) at 0°, 140° W (Fig. 2a). (The TIW KE is calculated as 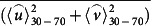, where *u* and *v* are the eastward and northward components, respectively, of velocity observed by TAO moorings, 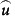 and 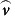 are the 12–40 days band-pass filtered, 〈 〉_30–70_ denotes the vertical mean over −70 to ∼−30 m and 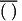 denotes a 40-day low-pass filtering.)

The results strongly indicate the modulating effect of TIWs on the occurrence of deep-reaching overturning in the pycnocline. Given that overturning and mixing usually accompany with each other, the result not only confirms the notion that TIWs lead to enhanced turbulence and mixing in the upper part of the thermocline^13^ but also implies that the modulation effects of TIWs on mixing can reach deeper depths of the pycnocline. (As density here is dominated by temperature^32^, in the remainder of the study we focus on the thermocline instead of the pycnocline.)

**Figure 3:**
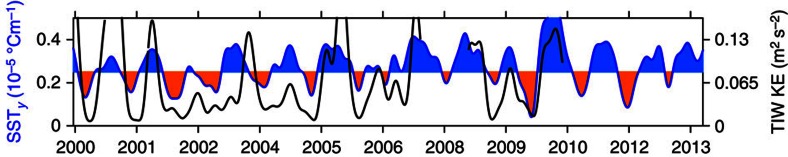
Separating TIW and non-TIW periods with meridional SST gradient. The blue curve denotes the meridional SST gradient (SST_*y*_). The TIW (non-TIW) periods are depicted with blue (red) shading. The black curve denotes the 140-day low-pass-filtered TIW KE. The correlation coefficient between the filtered TIW KE and the SST_*y*_ over 2000–2010 is *r*=0.47, with *P*<0.001 and 95% CI=(0.44, 0.49).

In the following, we will further verify the impact of TIWs in enhancing the occurrence of overturns by comparing overturn properties of TIW periods with those of non-TIW periods. To this end, we followed ref. 44 and defined TIW periods and non-TIW periods based on meridional sea surface temperature (SST) gradient. The reason why we adopted this strategy is because a large portion of the fine resolution Argo profiles and overturns are found between the years 2011 and 2014, whereas the TAO velocity measurement and hence the TIW KE index are not available since 2011. SST^45^ (see Methods for data source) is first averaged in longitude spanning 12° centred at two latitudes of 140° W, 4.5° N and 0.5°N, and then the averaged SSTs are 140-day low-pass filtered; finally, the meridional gradient of the filtered SSTs (SST_*y*_) is calculated. TIW periods are defined as the periods when the SST_*y*_ is >0.25 × 10^−2^ °C km^−1^; other periods are defined as non-TIW periods (Fig. 3). This proxy matches the TIW KE index well (Fig. 3).

Overall, about two-thirds of the total time periods belong to TIW periods and one-third of them belong to non-TIW periods (Fig. 3). The numbers of overturns within 160°–110° W and 3° S–6° N over the years 2008–2013 are 314 and 67, whereas the numbers of fine-resolution Argo profiles are 4,952 and 1,783, in TIW and non-TIW periods, respectively. This leads to the occurrence probability of 6.34% for TIW periods (the 95% bootstrap confidence interval (95% CI)=(5.67%, 7.07%)) and of 3.76% for non-TIW periods (the 95% bootstrap CI=(2.92%, 4.71%)). The former is 69% larger than the latter (Fig. 2d); in addition, the occurrence probability for TIW periods is larger at almost every depth than non-TIW periods within the upper and centre of the thermocline (depths −0.5 to ∼1; Fig. 2c). The results demonstrate again that TIWs are associated with a higher occurrence of overturns.

### Link the overturns with TIWs via shear instability

The observed higher occurrence of deep-reaching overturns during TIWs, so far established in the seasonal and period-to-period cycles, calls for a physical interpretation. The overturns are indicative of breaking of internal waves and/or turbulence generated by shear instability. Two ways may be employed to demonstrate this physical interpretation. One is the LSA, which can determine the potential instabilities of an observed flow by providing locations and other detailed properties of the exponentially growing unstable modes (see Methods and refs 18, 40, 41, 42). The LSA is applied to ∼8 × 10^4^ hourly TAO profiles of years 2000–2010 at 0°, 140° W (this site locates meridionally at the centre of Pacific TIWs and thus is representative for TIW studies^21^).

**Figure 4:**
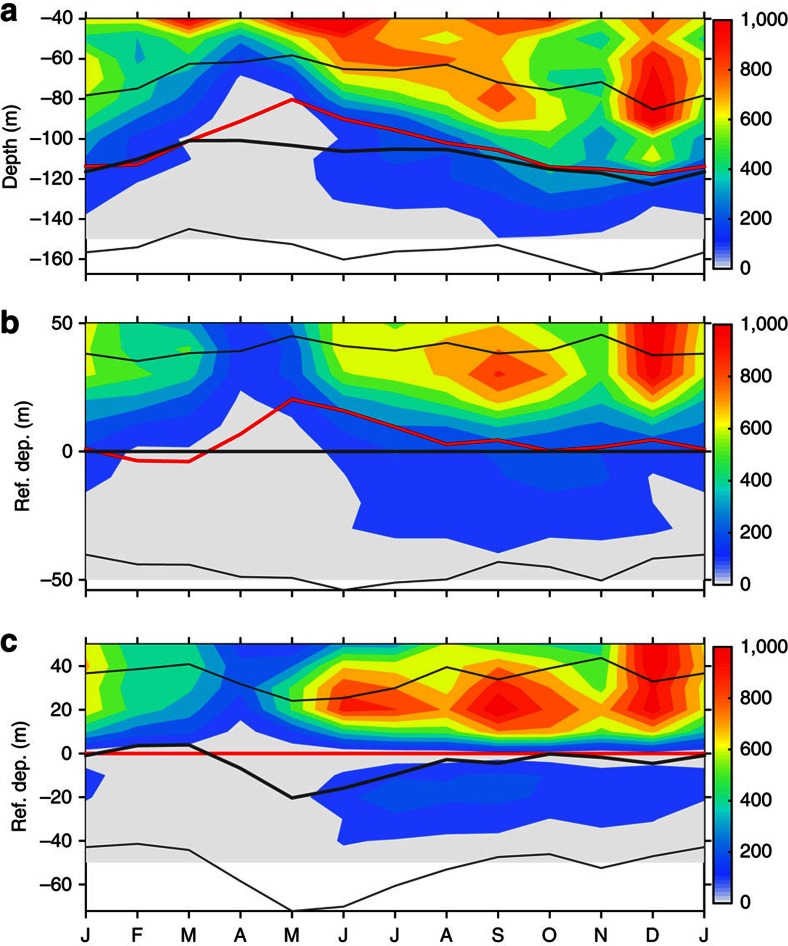
Monthly climatology of count of critical levels at 140° W of the Equator. (**a**) On physical depths. (**b**) On depths that is referenced to hourly centres of the thermocline (defined as the depth of maximum vertical gradient of 40-m running-averaged temperature). (**c**) On depth that is referenced to hourly centres of the EUC (defined as the depth of maximum eastward velocity). Shown are counts in 10-m bins. In each panel, the red curve denotes the average depth of EUC core, the black thick curve denotes the average depth of the thermocline centre and the two black thin curves denote the average upper and lower bounds of the thermocline.

The monthly counts of the potential instabilities (in terms of the critical levels of the detected unstable modes; see Methods) is shown on physical depth in Fig. 4a and on referenced depths in Fig. 4b,c. In Fig. 4b, the depth is referenced to the thermocline centre of each profile, which is defined as the depth of maximum vertical temperature gradient (before calculation, temperature is 40-m running smoothed, to remove effects of noises and intermittent waves). In Fig. 4c, the depth is referenced to the EUC core of each profile, which is defined as the depth of maximum eastward velocity. In addition, the long-term averaged monthly depths of the EUC core, the thermocline centre and the upper and lower thermocline bounds (defined as the depths of half the maximum vertical temperature gradient), are overlaid on the three panels of Fig. 4.

A prominent feature is the seasonal variation of the potential instabilities (Fig. 4a). Within the thermocline, more potential instabilities were found in boreal summer to winter, while relatively few potential instabilities were found in boreal spring. There were also fewer potential instabilities occurring during October and November. This feature is consistent with the seasonal variations of both TIW KE and occurrence probability of Argo-determined overturns (Fig. 2a,b).

Another distinguished characteristic is the deep-reaching nature of the potential instabilities. From boreal summer to winter, the potential instabilities may reach down to −120 m, with deepest depths of −150 m in fall. Potential instabilities occurring below the upper thermocline bound are as many as those above it. As the depth of the upper thermocline bound varies from −80 to −60 m and roughly coincides with the top of the observed upper core layer^13^, it demonstrates that the upper core layer mixing is a remarkably persistent phenomenon in the study site. Moreover, ∼15% of all the determined instabilities locate well below the average centres of both the EUC and the thermocline, which coincide with each other in boreal June to March (Fig. 4a). By contrast, in spring, instabilities can only occur within the upper 75 m, which is ∼25 m above the thermocline centre.

**Figure 5:**
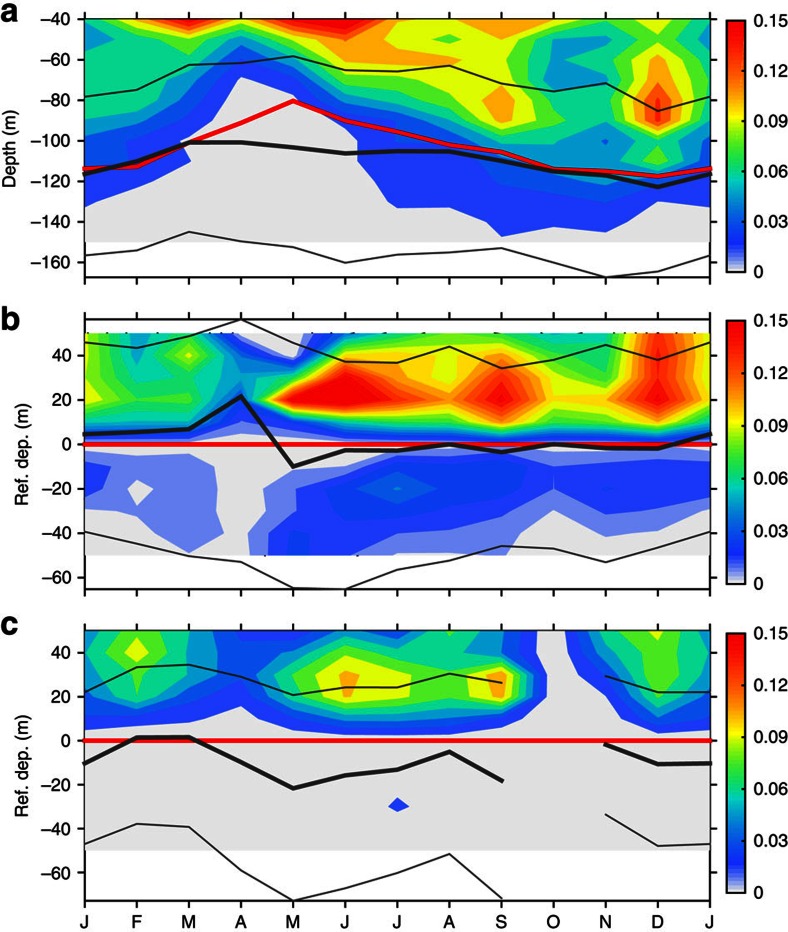
Monthly climatology of occurrence probability of critical levels at 140° W of the Equator. (**a**) On physical depths. (**b**) For periods of TIWs but on the depth that is referenced to instantons EUC cores (see caption of Fig. 4). (**c**) The same as in **b**, but for periods of non-TIWs. TIW (non-TIW) periods are defined when the TIW KE is larger (less) than 0.04 m^2^ s^−2^. The occurrence probability is defined as the ratio of the number of unstable modes over the number of profiles in 10-m bins. The red curve denotes the average depth of EUC core, the black thick curve denotes the average depth of the thermocline centre and the black thin curves denote the average depths of the thermocline bounds.

As the numbers of hourly profiles in each month and at each depth are nearly the same, the occurrence probability of the instabilities (Fig. 5a) displays a similar pattern as the counts of potential instabilities shown in Fig. 4a.

When all the potential instabilities are redistributed on the depth that is referenced to the thermocline centre of each profile (Fig. 4b), a centre of potential instabilities emerges ∼26 m above the thermocline centre (the averaged thickness of the upper flank of the thermocline is ∼40 m), in particular in summer to fall. The centre coincides well with the peak at 0.7 on the normalized coordinate of the Argo-detected overturns (Fig. 1d). In addition, potential instabilities occur also in and below the centres of the thermocline. Alternatively, when the potential instabilities are redistributed on the vertical coordinate that is referenced to the EUC core of each profile (Fig. 4c), a striking feature is clearly observed: in addition to those in the upper core layer, an isolated region of potential instabilities stand out in the lower flank of the EUC. These potential instabilities occur only in summer to winter and accounts for ∼20% of those in the upper core layer. Instabilities peaked ∼20 m above and below the EUC core, but no instabilities were found in the EUC core.

The relation of the deep-reaching potential instabilities to TIWs is further illustrated from a period-to-period point of view in Fig. 5b,c. In these two panels, we show the occurrence probability of potential instabilities in the TIWs and non-TIWs periods, respectively, on the depth referenced to the EUC core. Here, the TIW periods are defined as periods when the 140-day low-passed TIW KE (Fig. 3) is larger than 4 × 10^−2^ m^2^ s^−1^ (corresponding to a characteristic horizontal velocity of 20 cm s^−1^); the other periods are defined as non-TIW periods. These newly defined periods of TIWs and non-TIWs are consistent with those defined based on the SST gradient (Fig. 3).

The occurrence probability is ∼50 to ∼100% larger, almost at every depth during TIW periods than non-TIW periods in the upper core layer (except in February). In particular, the instabilities of the lower flank of the EUC can only occur with the existence of TIWs. Consequently, the results clearly demonstrate the enhancement effect of TIWs on the occurrence of potential instabilities in both the upper and lower flanks of the EUC.

### Link the instabilities to low Richardson numbers

**Figure 6:**
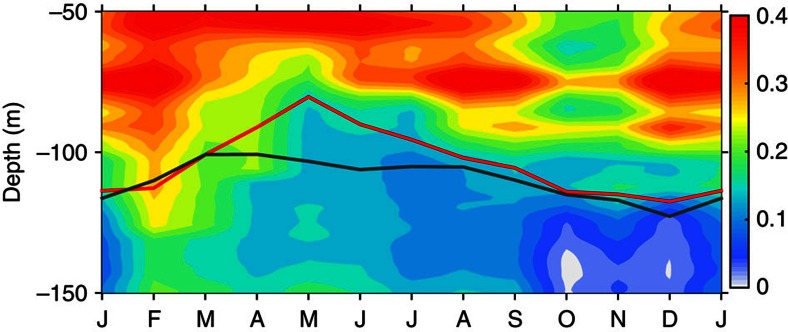
Monthly occurrence frequency of low Richardson number. The occurrence frequency is calculated as the ratio of numbers of Ri≤0.35 over numbers of all Ri in 10-m bins. The red curve denotes the average depth of EUC core and the black curve denotes the average depth of the thermocline centre.

The other way to link the shear instability to the deep-reaching feature of the detected overturns in the Argo profiles (as well as the TAO-determined potential instabilities) is to examine the Richardson number, Ri (*=N*^2^*/S*^2^, where 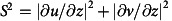 is the shear squared). The shear instability (in particular of the Kelvin–Helmholtz type) is dynamically related to the local Richardson number, a critical value of which Ri_c_ is ∼0.25. Ri=Ri_c_ is an equilibrium state for turbulence in a stratified shear flow. When Ri<Ri_c_, turbulence may be initiated or continue to grow due to shear instabilities. When Ri>Ri_c_, the flow is dynamically stable and any turbulence will decay^46^,^47^; however, when Ri is close to Ri_c_, the flow may lie in the regime subject to marginal instability^48^. For example, turbulence can persist up to a Ri value typically near 1/3 (ref. 49). The marginal instability is well identified in the upper layer (upper ∼75 m) of TIW periods at 0°, 140° W^50^. Accordingly, based on the hourly TAO measurements over the years 2000–2010, the occurrence frequency of Ri≤0.35 was computed to represent the possibility of instabilities (Fig. 6).

The high occurrence frequency of Ri≤0.35 is roughly associated with the high-occurrence probability of potential instabilities as shown in Fig. 5a, although they match well only in their main structure, rather than in details. The pattern of higher occurrence frequency (say ≥0.25) includes a deep extension to ∼−100 m in winter and summer months, and a subsurface centre (at −50 m) from February to September. The lower bound of the higher-occurrence frequency is generally confined to the centres of the thermocline and the EUC. This feature is consistent with the occurrence of the potential instabilities.

**Figure 7:**
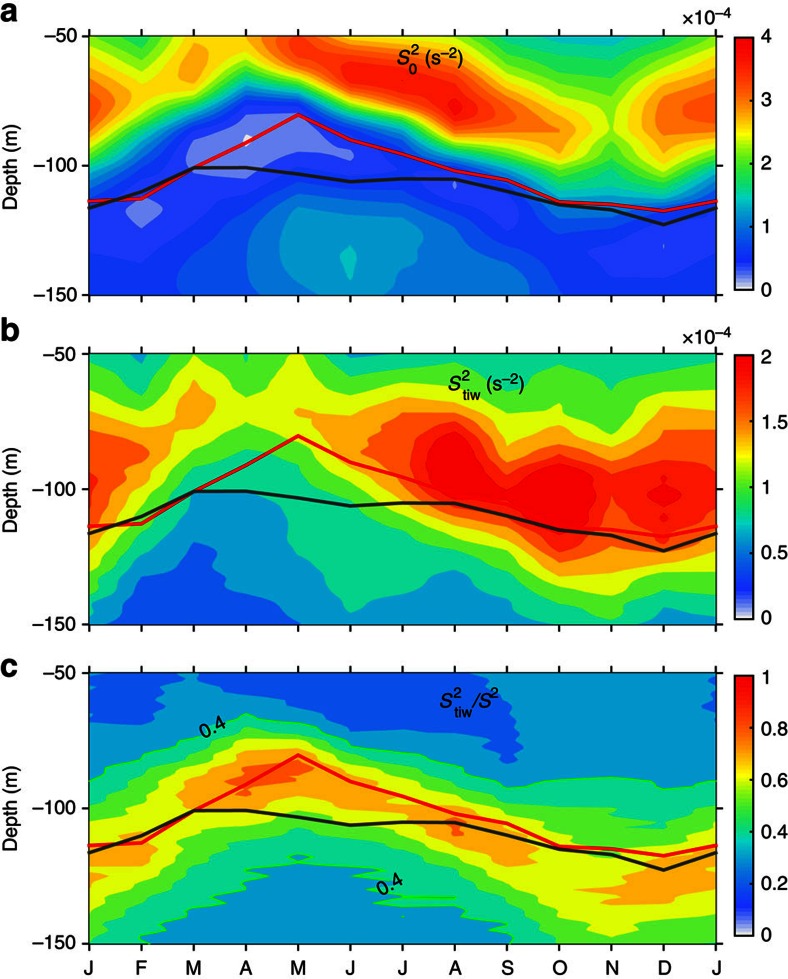
Shear of background flows and TIWs. (**a**) Shear squared induced by the background flow, 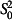. (**b**) Shear squared associated with TIWs, 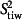. (**c**) The proportion of the shear squared associated with TIWs, 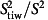. In **a**,**b** and **c**, the red curve denotes the average depth of the EUC core and the black curve denotes the average depth of the thermocline centre. In **c**, contour of 0.4 is highlighted for reference. The different colour scales in **a** and **b** are noteworthy.

The inconsistence in detailed structures between the occurrence frequency of Ri≤0.35 and the occurrence probability of potential instabilities is explainable. In particular, from February to June, relatively high occurrence frequency of Ri≤0.35 is found below −100 m, where fewer potential instabilities were determined here (Fig. 5a). This may be because the shear in the depths is weak (Fig. 7). Hence, although a large portion of Ri is small (resulted from weak stratification), there was not enough KE available in the mean flow to drive unstable modes that have high-enough growth rate^18^ that could pass the growth rate criterion used in the LSA (see Methods).

As mentioned, the annual cycle of the occurrence frequency of low Ri should have resulted from not only the shear of EUC and the TIWs, but also the thermal structure of the upper ocean. All the processes and properties are ultimately also related to wind stresses and exhibit seasonal variations. For example, in boreal spring the wind reduces, the shear weakens and the water warms with increased stratification in the subsurface layers; since late summer, the wind stress increases, the shear strengthens and the surface water cools down with decreased stratification.

Nevertheless, the contribution of TIWs to the low Ri and therefore the generation of potential instabilities could be roughly isolated from the EUC. This was done by separating the individual shear they induce. The shear squared induced by the background EUC is calculated as 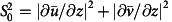, where 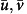 are the 40-day low-pass-filtered velocities, representing the background flows, whereas the shear squared associated with the TIWs is estimated as the difference between the original and the background shear squared: 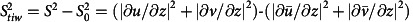 (Fig. 7a,b). In general, the EUC is associated with stronger shear squared, which centres ∼20–50 m above the seasonally varying EUC core (Fig. 7a), whereas the TIWs are associated with weaker shear (Fig. 7b).

However, the magnitude of TIW-induced shear squared could reach half of that induced by the EUC in a thick layer. In addition, as a prominent feature, the TIW-induced shear is centred just above the EUC core and covers both the upper core layer and the layers immediately below the EUC core, in particular during TIW seasons (boreal summer to winter). The TIW shear covering the EUC core adds to the EUC-induced shear and provides the conditions favourable for instability; besides, the strong velocity of TIWs provides necessary KE for the instability to grow fast. This explains the occurrence of potential instabilities occurring below the centres of both the EUC and thermocline (Fig. 4).

The portion of the TIW-induced shear is calculated as 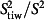 (Fig. 7c). The TIW-induced shear accounts for 30∼50% for most of the upper layer. This percentage is consistent with the direct measurements that shows ∼30% larger of shear induced by TIW^13^. In particular, it accounts for 60∼80% just above and below the EUC core. These results indicate that the TIWs provide a modulating effect on the generation of unstable disturbances.

### TIWs and instabilities at ENSO timescales

**Figure 8:**
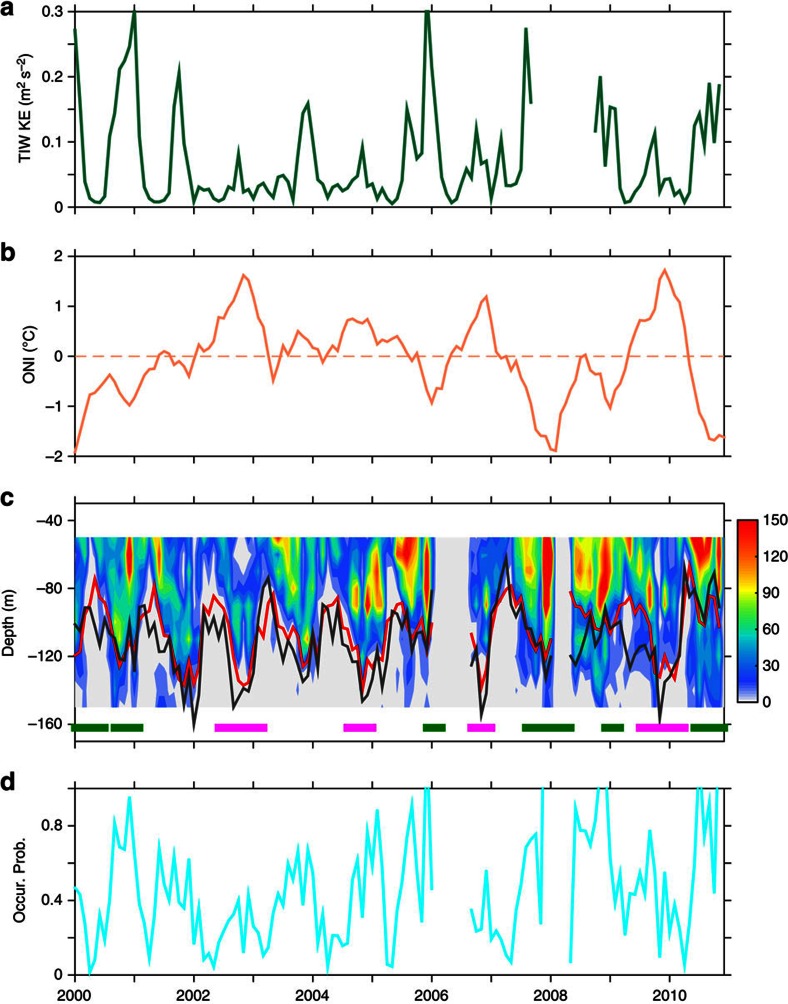
Relation among instabilities, TIWs and large-scale processes on the inter-annual time scale. (**a**) Monthly TIW KE. (**b**) ONI, showing the Niño 3.4 (5° S–5° N, 170–120° W) SST anomaly (1981–2010 mean removed) calculated from v2 of the Optimum Interpolation Sea Surface Temperature (OISST). The dashed line denotes zero. (**c**) Monthly count of unstable modes in 10-m bins. The green and magenta bars on the bottom denote periods of the La Niña and El Niño conditions, defined when the ONI is ≤−0.5 °C and ≥0.5 °C, respectively. The red curve denotes the average depth of EUC core and the black curve denotes the average depth of the thermocline centre. (**d**) Monthly occurrence probability of unstable modes, defined as the ratio of counts of critical levels occurring between −50 and −150 m of a month over the number of profiles of the given month.

In Fig. 8a,d we show the monthly TIW KE and the occurrence probability of unstable modes within the thermocline (−50 to ∼−150 m) for the years 2000–2010. In years of stronger TIWs, larger occurrence probability of unstable modes are observed. The high correlation between them (correlation coefficient *r*=0.71, *P*-value<0.001, 95% CI=(0.62, 0.79)) further demonstrates that TIWs are associated with higher thermocline instability occurrence also at the inter-annual timescale.

Previous studies, based on modelling results and a TIW proxy in terms of the SST variance, found that the activity of TIWs is larger under La Niña conditions and smaller under El Niño conditions, because the former are associated with stronger latitudinal gradient of SST immediately north of the Equator and thus more occurrence of baroclinic instability^51^. Using the monthly TIW KE and the Oceanic Niño Index (ONI) calculated from the monthly Optimum Interpolation Sea Surface Temperature (ref. 52) (Fig. 8a,b), we confirmed such a significantly negative correlation (correlation coefficient *r*=−0.69, with *P*-value<0.001 and 95% CI=(−0.77, −0.59)).

The implication of the relation is that the inter-annual variation of occurrence probability of instabilities could also be related to El Niño and La Niña conditions. The correlation coefficient between the ONI and the occurrence probability (Fig. 8d) is −0.58, with *P*-value<0.001 and 95% CI=(−0.68, −0.45). It implies that there were more potential instabilities, associated with more TIWs, under La Niña than under El Niño conditions.

Moreover, the extension range of potential instabilities differs between two conditions (Fig. 8c). Under El Niño conditions, the potential instabilities are mainly confined to the upper flank of the thermocline, except for stronger TIWs. By contrast, under La Niña conditions, the potential instabilities mostly can reach to the lower flank of the thermocline.

## Discussion

In the present study, we show the existence of overturns in the deep depths of the thermocline. We also show that the potential instabilities are organized in a physically quite reasonable structure. Given the good coincidence of the determined potential instabilities and the measured mixing during November 2008 (Supplementary Figs 3 and 4), it is anticipated that the potential instabilities during other time are also associated with mixing, although the mixing intensity and accompanying heat fluxes can not be correctly estimated yet.

In the cold tongue of the equatorial Pacific, maintaining cool SSTs in the presence of intense solar heating requires a combination of subsurface mixing and vertical advection to transport surface heat downward^43^,^53^,^54^,^55^,^56^. Analyses of direct turbulence measurements have demonstrated that the subsurface mixing (over −60 to ∼−20 m) reduces SST during a particular season—boreal summer^43^. If mixing is indeed associated with the detected overturns and potential instabilities in the deep depths, it could also blend water between the upper part and middle/lower part of the thermocline, resulting in cooling of the upper thermocline, and further cooling of the surface.

The TIW-related mixing during La Niña conditions may have rich implications for ENSO dynamics. It has been found that incorporating TIWs in the ocean–atmosphere coupled models results in a significant asymmetric negative feedback to ENSO^57^,^58^,^59^ (anomalously heating the Equator under La Niña conditions and cooling it under El Niño conditions via horizontal advection). Accordingly, the asymmetric negative feedback is argued to explain the observed asymmetric feature of a stronger-amplitude El Niño and weaker-amplitude La Niña relative to the models. However, the cooling effect via vertical mixing associated with TIWs, in particular during La Niña conditions, was missed or under-represented by the numerical models due to underestimation by the parameterizations^60^. Therefore, the effects of TIWs on ENSO development requires to be re-examined.

To best simulate the oceans, numerical models need to reproduce or properly represent the TIWs and the associated turbulence. Although the main structure of TIWs can be reproduced in some coarse resolution ocean general circulation models (OGCMs)^30^, the small-scale structures of the frontal areas of TIWs, which are key regions of turbulence generation^28^,^29^,^61^,^62^, remain unresolved by coarse resolution OGCMs and ocean-atmospheric coupled models. This shortage may lead to underestimates of thermocline mixing by the oversimplified vertical mixing parameterizations incorporated in coarse resolution OGCMs^60^ and hence to model-data deviations not only in the equatorial ocean but also in mid-latitudes^63^,^64^.

## Methods

### Data processing

The Argo data (see below) covers the period from 2000 till June 2014 and only profiles with vertical sample resolution of at least 2 m and with maximal sampling depth deeper than −200 m are used to detect overturns. Both hourly temperature and velocity from TAO mooring at 0°, 140° W is interpolated (extrapolated if needed) at 1 m spacing grids for both Ri calculation and the LSA (see below). As salinity observations of TAO are not sufficient and their contribution to density in this region is minor, salinity needed for real-time density calculation was often replaced by its temporal average^50^; here we use the salinity climatology averaged from 251 Argo and CTD (CTD is obtained from the same source of Argo) profiles falling into the range of 0°±0.5°, 140° W±0.5°.

### The Thorpe method

The Thorpe method^38^ is commonly used for estimating dissipation rate and vertical turbulence diffusivities, which is based on the size of detected density overturn patch and the stratification intensity over the patch, of a measured potential density profile. In this work the Thorpe method is applied only for overturn detection, rather than diffusivity estimation (because the resolution of the Argo profiles is still too low for such estimation). The Thorpe method is suitable to be applied in the thermocline where the stratification is strong; in contrast, care should be taken in layers of low stratification due to its sensitivity to noise^65^; therefore, both the upper and lower layers of low stratifications defined by 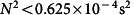 are omitted from our analysis. The size of any overturn should not be smaller than three times the profile resolution, a minimum criteria for the overturn size^66^. Profiles with large spikes from any of the properties (temperature, salinity and pressure) were removed from the data set. With the above criteria, few unreasonably large overturns are still found (see Supplementary Fig. 2). Therefore, each detected overturn and the corresponding density profile were carefully examined afterwards, to guarantee that it is physically sensible. In particular, the detected overturns that have sizes >30 m were removed.

### Linear stability analysis

The LSA is designed to detect instabilities potentially occurring in an observed flow. The stability of an inviscid, incompressible, stratified, unidirectional shear flow to small disturbances is determined by the solutions of the Taylor–Goldstein equation:


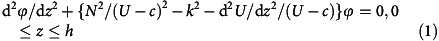


where *U(z)* and *N(z)* are the profiles of *z*-dependent mean velocities and buoyancy frequency, respectively, and 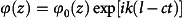 is the *z*-dependent stream function of a disturbance with real horizontal wavenumber *k* and complex phase speed *c*=*c*_*r*_+*ic*_*i*_. Here, *ϕ*_0_(*z*) is the amplitude of the stream function, *t* is time and *l* is along the direction of the perturbation wave vector. For non-parallel flows, the stability can be examined by taking *U* as the velocity component in the direction (*α*) of the disturbance wave vector: 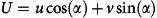, where (*u*, *v*) is the measured eastward and northward components of the velocity vector.

The Taylor–Goldstein equation (equation (1)) is a linear eigenvalue problem that can be solved numerically using matrix method^40^,^41^,^42^,^67^ subject to prescribed boundary conditions. In this study, zero condition is applied at both surface (*z*=0) and the lower boundary *z*=−200 m. Unstable modes should at least have the property *kc*_*i*_>0, to ensure that the disturbance grows exponentially. Useful quantities derived through solving the problem include the following: the wave vector direction of the instability perturbation wave, *α*; the perturbation wave length, 2π/*k*; the horizontal phase speed of the perturbation wave, *c*_*r*_; the growth rate of the perturbation wave, *kc*_*i*_ and the critical level of it, *z*_*c*_, which is defined as the depth where *U*(*z*_*c*_)−*c*_*r*_=0; the critical level is also considered as the position of the unstable mode.

Before applying the LSA, the data are carefully processed. The hourly TAO velocities are interpolated to a 1-m grid using cubic splines from surface to −200 m. Specifically, in the upper layers where velocity data are not available (the upper −25 m or −37.5 m, depending on data quality), the profiles of *u* and *v* are extrapolated using a polynomial fit, which should meet the requirement that the first derivative is continuous between −200 m and the surface, and approaches zero at the top two grids (−1 m and 0).

The hourly temperature of TAO observations is inter/extrapolated into the same 1- m spacing grids. As the temperature sample spacing is sparse (see Supplementary Fig. 3), a special strategy is applied. First, the raw data are interpolated into 5-m (above −50 m), 10-m (between −50 and −150 m) and 50-m (between −150 and −500) grids using a linear method. Second, the inter/extrapolated data are further inter/extrapolated into the 1-m resolution grids using a cubic spline method (other grid spacing and inter/extrapolated methods are tested; Supplementary Table 1). If data at the lowest sample grids (−300 m and −500 m; sometimes also at −180 m) are not available, their first derivatives are required to smoothly approach climatologic values, a similar manner as used in dealing with (*u*, *v*) on the top grids. We found that if the unavailable temperature data in the lowest sample grids are not constrained by prescribed values (such as by nudging their first derivatives to climatology), extrapolation may produce unphysically extreme values out of the data ranges and lead to obviously artificial unstable modes. Apart from this, the LSA results seem only weakly sensitive to grid spacing and inter/extrapolation methods (see Supplementary Fig. 3 and Supplementary Table 1). Nevertheless, all detected unstable modes that are below −150 m are rejected for accuracy.

Salinity measurements are sparse, so a temporal and spatial mean that is averaged into the 1-m grid over all fine resolution Argo measurements over the region of 0°±0.5°, 140° W±0.5° is employed instead. This substitution of salinity does not induce problems in *N*^*2*^ calculation and in the LSA, because the effect of salinity on density is small^32^. A similar treatment is adopted in ref. 50 at the same location.

As no prior information of the perturbation waves exist, the LSA scans both the wave vector directions and the wave numbers for each observed flow. For computational efficiency, the disturbance wave vector direction *α* is scanned from 0° to 180° with interval 15° (direction 0 represents east) in this study (direction 0 to ∼−180° is symmetry to 0 to ∼180° and need not be scanned); the hourly mean component of TAO velocities (*u*, *v*) at *α* direction, that is, *U*, is calculated subsequently. The wave number *k* of the perturbation wave is scanned over 85 values ranging from 2*π*/60 to 2*π*/1,000 m^−1^. For a given wave vector (*k*, α), the number of unstable modes, which are determined based on criteria of ref. 18 (see below), ranges from 0 to ∼10. Furthermore, all potential unstable modes of the flow (in terms of critical levels) constitute several mode families.

The idea of mode family is based on the nature that the critical level *z*_c_ of the flow is relatively consistent, even though most mode properties profile vary with *k* and α. A histogram of *z*_c_ obtained from all (*k*, α) vectors is constructed and peaks identified. All modes close to a given peak (that is, having critical levels between the adjacent minima of the histogram) are considered part of the same mode family, which usually focus in different depth ranges^16^,^18^ (Supplementary Fig. 3). For each mode family, the fastest growing mode (should satisfy additional criteria; see below) is defined as the unstable modes of the flow (Supplementary Table 1 and Supplementary Fig. 4).

The criteria (see ref. 18) adopted to determine all possible unstable modes (which are in terms of critical levels) and reasonable mode families include cutoff depths, cutoff growth rate of instability, cutoff wavelengths, Ri criteria, critical layer criteria and others, which are described in details below.

The cutoff depths are −40 m and −150 m in the present study. This criterion rejects potentially unphysical modes that are induced by extrapolation near the boundaries as mentioned above. Previous sensitivity studies also demonstrated that the location of detected unstable mode near boundary may depart to some extent from its real position^42^.

The cutoff growth rate of instability is 1 h^−1^. This criterion guarantees that the instability grows faster than hourly variation of the mean flow.

The cutoff wavelengths depend on the sampling spacing of the temperature measurements. Instabilities of inviscid, non-diffusive, stratified shear layers typically have wavelength around 2*π* times the thickness of the shear layer^18^,^41^,^68^. Based on this, modes with wavelength 65 m are likely to grow from layers of thickness 10 m, the Nyquist wavelength of the ∼5-m vertical bins of TAO temperature data in the above −60 m depth; similarly, modes with wavelength 250 m (500 m) are likely to grow from the ∼20-m (∼40cm) vertical bins of TAO temperature data over −60 to ∼−140-m (−140 to ∼−200) depths. We remove modes of wavelengths <65 m above −60 m depth and of wavelengths <250 m between −60 and −140 m, and of wavelengths smaller than 500 m between −140 and −200 m, to avoid possibly unphysical modes that are resulted from interpolation. The bins of TAO velocity data are constantly 5 m and thus do not require extra limitations of wavelengths.

The lowest Ri of the profile should be <0.25. Moreover, in the vicinity (±1/14 wavelength) of the critical level of the potential unstable mode, lowest Ri is required to be <0.25, to assure typical Kelvin–Helmholtz instability of this unstable mode^17^.

Any mode family must include at least one resolved mode with a larger wavelength than the fastest growing mode and at least one with smaller wavelength. This effectively rejects modes whose true maxima lie outside the range of wavelengths tested.

In addition, the critical level(s) is determined as the depth(s) where |*U*(*z*)−*c*_r_|≤0.01 (m s^−1^) in the present study. Why we added such a criterion is because the 1-m spacing, inter/extrapolated velocity profile is still discrete so that it may not guarantee a depth that meets the restrict definition of critical level: *U*(*z*)−*c*_r_=0. Under such a criterion, there may exist more than one critical level of a (*k*, α, *c*_r_) vector that satisfy the above criteria. All are retained for further analysis.

(It is noteworthy that the LSA performed here differs in physics from ref. 18 in that we did not include effect of eddy viscosity in equation (1), while the referred work did. In ref. 18, the authors demonstrated that the addition of eddy viscosity to equation (1) damped the generation of instabilities mainly at night when turbulence is strongest. However, the effect of eddy viscosity could be subtle under different conditions, that is, it may also destabilize a stratified shear flow^69^.)

Supplementary Figs 3 and 4 show detailed results of LSA that is applied to an example profile and to consecutive profiles over a period of ∼8 days, respectively. Based on the flow shown on Supplementary Fig. 3, we also discuss the sensitivity of the LSA to the inter/extrapolation method (Supplementary Table 1). In Supplementary Note 1 we describe the details of both analyses. In summary, the sensitivity study suggests that the unstable modes occur in vicinity of low Ri, and as long as this region of low Ri is accurately solved and not close to the boundary, reasonable unstable mode can be detected. By Supplementary Fig. 4, we demonstrate the usefulness of LSA via showing the coincidence of the detected unstable modes with the direct turbulence measurements.

### Data availability

Argo and CTD data are obtained from the Coriolis project (http://www.coriolis.eu.org). These data were collected and made freely available by the International Argo Program and the national programmes that contribute to it. TAO mooring data are available at http://www.pmel.noaa.gov/tao/data_deliv. SST used for calculating the meridional SST gradient (Fig. 3) is daily Reynolds analysis^45^ with horizontal resolution 0.25° × 0.25°, provided by the National Oceanic and Atmospheric Administration and downloaded from the Integrated Climate Data Center at http://icdc.zmaw.de. SST used for calculating the ONI is v2 of the Optimum Interpolation Sea Surface Temperature, available at http://www.cpc.ncep.noaa.gov/data/indices/sstoi.indices. Velocity data used for plotting the mean depth of the EUC core (Fig. 1b; blue curve) is provided by G.C. Johnson via http://floats.pmel.noaa.gov/gregory-c-johnson-home-page.

## Additional information

**How to cite this article:** Liu, C. *et al*. Deep-reaching thermocline mixing in the equatorial pacific cold tongue. *Nat. Commun.* 7:11576 doi: 10.1038/ncomms11576 (2016).

## Supplementary Material

Supplementary Information Supplementary Figures 1-4, Supplementary Table 1, Supplementary Note 1 and Supplementary References.
