# Supplementary material for: Deep-reaching thermocline mixing in the equatorial pacific cold tongue
Source: Nat Commun. 2016 May 12;7:11576. doi: 10.1038/ncomms11576 (PMC4865879; doi:10.1038/ncomms11576)
Supplement: Supplementary Information — Supplementary Figures 1-4, Supplementary Table 1, Supplementary Note 1 and Supplementary References. [file ncomms11576-s1.pdf]

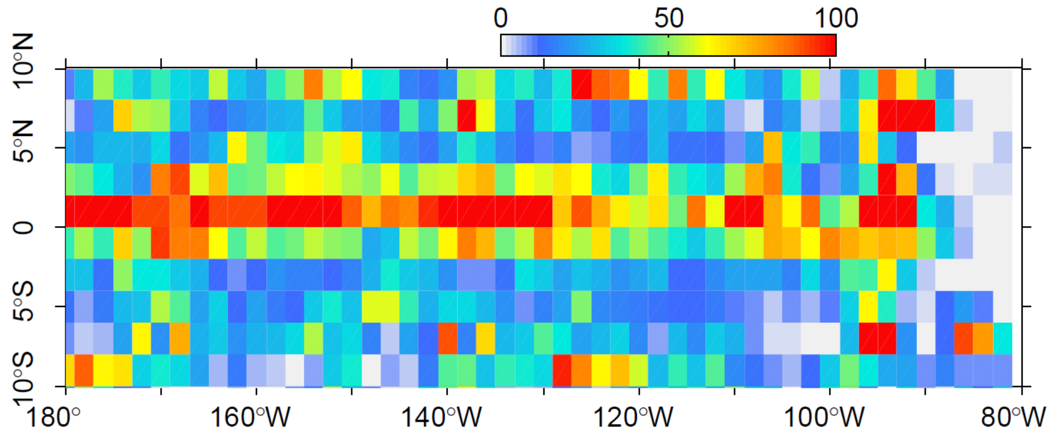

Supplementary Figure 1 | Distribution of fine resolution Argo profiles in the tropical Pacific Ocean. Color: numbers of fine-resolution (≤2 m) Argo profiles in each 2 °×2 °bin.

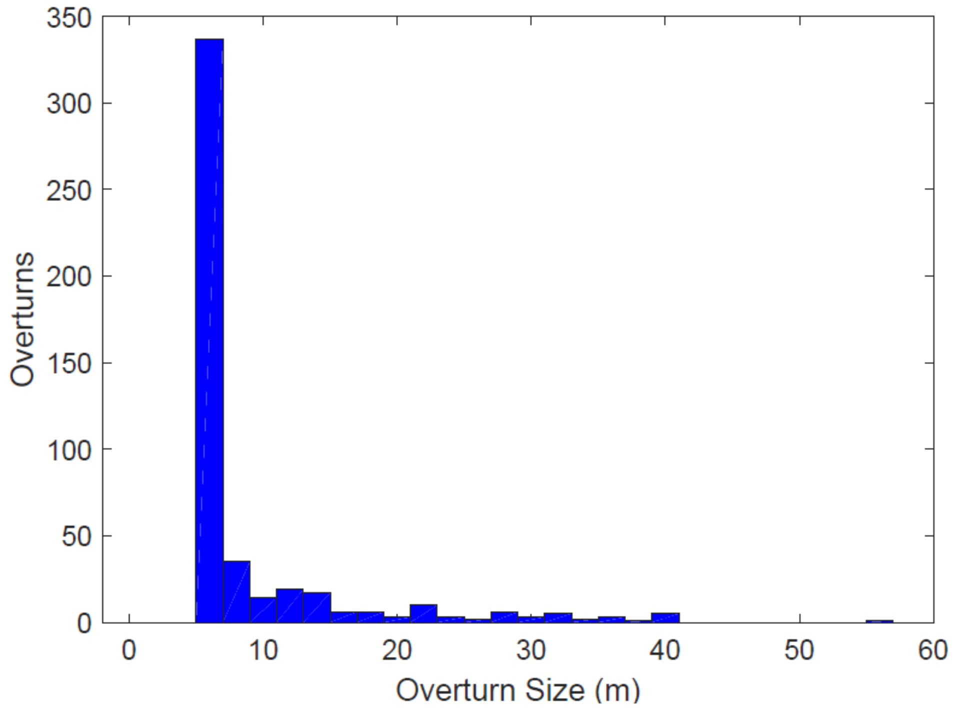

Supplementary Figure 2 | Histogram of sizes of overturns detected in-between 160~110° W, and 3° S~6° N. Most overturns have a size of 6 m, which is 3-grid spacing of the mostly 2 m-resolution Argo profiles; overturns larger than 30 m are rejected.

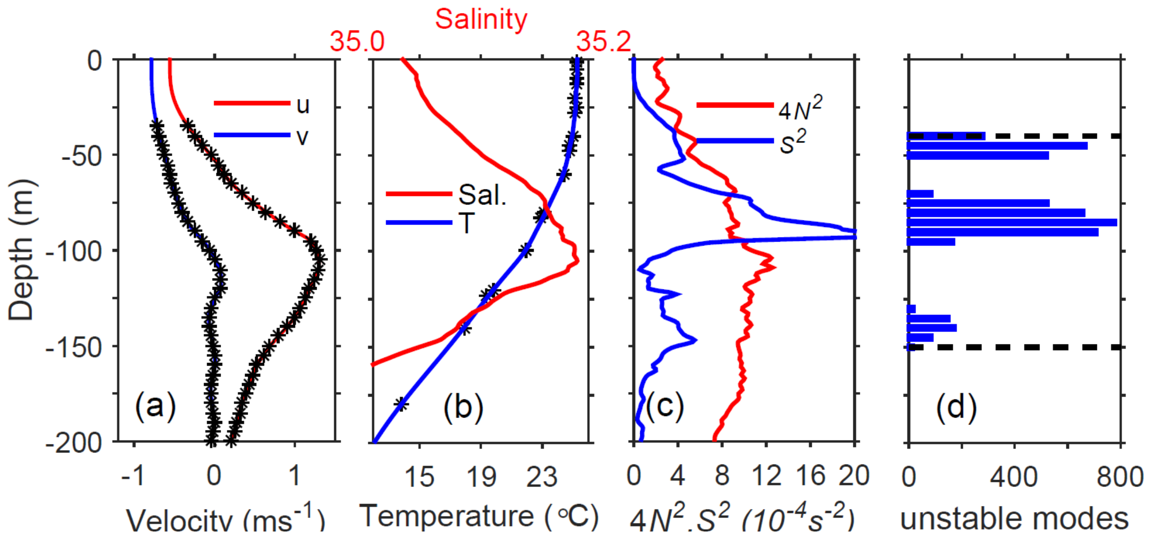

Supplementary Figure 3 | Linear stability analysis of a profile at 0º, 140ºW taken on 12:00, 3rd, November, 2008. a, The inter-/extrapolated, hourly mean eastward (*u*) and northward (*v*) velocity components. The black asterisks represent data provided by the Tropical Atmosphere and Ocean (TAO) measurement. **b**, The inter-/extrapolated hourly temperature and long term and spatially averaged salinity. The black asterisks represent temperature provided by TAO. **c**, Shear squared, S^2, and 4 times the buoyancy frequency squared, N^2,of the inter-/extrapolated flow. **d**, Unstable mode families. Thick dashed lines denote the depths of -40 m and -150 m, beyond which any detected unstable mode is rejected.

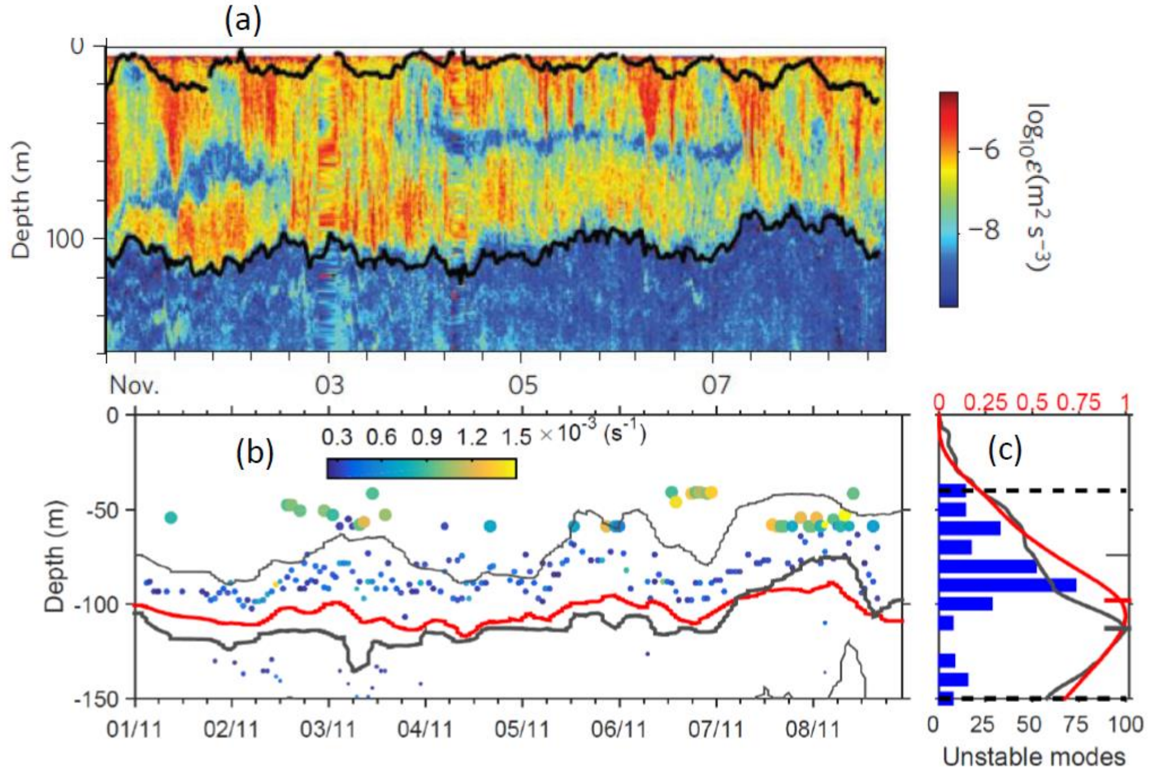

Supplementary Figure 4 | Time series of mixing at 0º, 140ºW in early November 2008. **a**, Color: turbulence dissipation rate, ε, obtained from direct turbulence measurements (reproduced from ref.[1]). The upper thick black curve represents the mixed layer base, while the lower thick black curve represents the core (eastward velocity maximum) of the Equatorial Undercurrent. **b**, Filled color circles: the critical levels of the LSA-determined unstable modes from the hourly TAO observations over the same period as **a**. Colors of the circles represent the growth rates (in s^{-1}) of the unstable modes, while sizes of the circles represent the wave numbers. The thick black curve represents the core of the Equatorial Undercurrent (EUC) determined from TAO; the thick red curve denotes the depths of the thermocline center (defined as the depth of the maximum vertical temperature gradient), while the thin black curves denote the bounds of the thermocline (most of the lower bound is below -150 m). **c**, Histogram of critical levels of the unstable

modes during this period. The black and red curves denote the temporally averaged vertical temperature gradient and the zonal velocity, respectively. Both of them are normalized so that they range between 0 and 1. Depths of the EUC core and the thermocline center are denoted at the right of the penal. The dashed lines at -40 m and -150 m denote the cutoff depths.

Supplementary Table 1 | Parameters of sensitivity study.

Cases	Inter/extrapolation method	Grid Spacing	Critical levels (m)	Ranges of corresponding mode families
1	cubic spline	1 m	-137, -92, -50	[-148 -128], [-98 -66], [-53 -47]
2	linear	1 m	-139, -90, -71, -48	[-150 -125],[-95 -85],[-83 -65],[-55 -40]
3	cubic spline	0.5 m	-139, -91.5, -49.5	[-150 -125], [-95 -65] , [-55 -40]
4	cubic spline	2 m	-136, -88, -72, -52	[-150 -124],[-98 -80],[-80 -60],[-58 -44]

Supplementary Note 1: Examples of linear stability analysis and sensitivity study

Supplementary Fig. 3d shows three detected mode families with the standard method (see Method) that is applied to the profile taken on 12:00, 3rd, November, 2008 at 0 °, 140 °W: one is peaked at -45 m, ranged between -53 and -40 m; the second is peaked at -85 m, ranging between -98 and -66 m; the third is peaked at -140 m, ranging between -148 and -128 m. The critical levels of the fastest growing disturbance of the three mode families are at -50 m, -92 m and -137 m, respectively. Note that the deepest critical level is below the Equatorial undercurrent (EUC) core, which is at ~ -100 m. Regarding the determined unstable modes, the wave vector directions are 15 °, 60 ° and 30 °, the wave lengths are 65, 350 and 580 m, and the growth rates are $(0.38h)^{-1}$, $(0.50h)^{-1}$ and $(0.86h)^{-1}$, respectively. All the 3 mode families occur in vicinities of lowest Ri. Compared with the direct turbulence measurement (Supplementary Fig. 4a), it proves that the determined three critical levels should be physical. For ease of description of the sensitivity analysis, we refer to this LSA experiment as Case 1.

In order to test the sensitivity of the LSA results to the inter/extrapolation for Ri calculation, three additional LSA experiments are conducted. The parameters of the cases are listed in Supplementary Table 1, which also includes the critical levels of the determined unstable modes and the vertical extension of corresponding mode families. In case 2, we use a linear method for inter/extrapolation. Case 2 almost produces the same depths of critical levels as Case 1, though it also produces an additional critical level at -71 m. The middle two families of Case 2 cover a range between -95 m and -65 m, similar to that of the middle mode family of Case 1, which is between -98 and -66 m. This

indicates that additional critical level in Case 2 seems to come from a sub mode family of the middle mode family of Case 1 (Supplementary Table 1). Case 1 is preferred in order not to induce unphysical modes (though in this selected profile, the additional critical level seems not artificial because it corresponds to a low Ri); by this way, we may also have missed some real unstable modes. We applied the same inter-extrapolation method as in Case 1 to finer and coarse grids in both Case 3 and Case 4, respectively. Case 3 produces 3 critical levels, with the lowest 5 meters lower than that of Case 1, and the other two almost at the same depths as Case 1. Case 4 produces 4 critical levels, similar to Case 2. In addition, the determined growth rates are all different from each other even from a same mode family.

The results suggest that, on one hand, the different inter/extrapolation method could determine the same large mode families; while, on the other hand, they may give slightly different critical levels. This is because the differences in both velocity and stratification caused by inter/extrapolation methods may alter the energy distribution of the flow, and result in both different locations and growth rates of the fastest growing mode. The variance of the critical levels among cases is within 5 meters, which won't change the final conclusions of the present work.

Supplementary Figs. 4b and c show the critical levels of detected unstable modes, accompanied with their growth rates and perturbation wave numbers, over the period between 1st and 9th, November, 2008 at 0°, 140°W, when direct turbulence measurements were conducted[1]. During this period, a TIW passed by and induced strong mixing in the upper part of the thermocline. A turbulent layer below the diurnal cycle layer (DCL) emerged since 28th, October, and became particularly prominent between

1st and 6th, Nov (Supplementary Fig. 4a). This layer was called the upper core layer (UCL), and was argued to be mechanically separated from the wind driven DCL, because a weak shear layer at around -50~-60 m existed and separated the upper and lower turbulent layers for ~10 days[2]. The instability in the UCL was attributed to the enhanced shear by the meridional velocity associated with the TIW[1]. We see that the detected critical levels are in the correct locations of mixing. It also shows that the growth rates of the instabilities are generally higher in the UCL than in the DCL (Supplementary Fig. 4b). Supplementary Fig. 4c displays the histogram of the determined critical levels over this period. It is seen that they peak at -60 m, -90 m and -140 m, with the lowest peak lies below the centers of both the temporally averaged EUC and thermocline, which are both around ~-110 m.

Supplementary reference:

1 Moum, J. N. *et al.* Sea surface cooling at the Equator by subsurface mixing in tropical instability waves. *Nat Geosci* **2**, 761-765, doi: 10.1038/Ngeo657 (2009).

2 Inoue, R., Lien, R. C. & Moum, J. N. Modulation of equatorial turbulence by a tropical instability wave. *J Geophys Res-Oceans* **117**, doi:Artn C10009Doi 10.1029/2011jc007767 (2012).
